# Management of dredged marine sediments in Southern France: main keys to large-scale beneficial re-use

**DOI:** 10.1007/s11356-024-33129-9

**Published:** 2024-04-15

**Authors:** Garry Dorleon, Sylvain Rigaud, Isabelle Techer

**Affiliations:** UPR 7352 CHROME, Laboratoire Géosciences de L’Environnement, Site Hoche - Université de Nîmes, 1 Place du Président Doumergue, 30000 Nîmes, France

**Keywords:** Dredged sediments, Physico-chemical characterization, Treatment and reuse, Waste management

## Abstract

Fifty million cubic meters of marine sediments are dredged each year in France in order to maintain harbor activities and sustain the economy of littoral territories. Because of anthropogenic activities in and around harbors, sediments can contain significant amounts of chemical and organic pollutants whose behavior during dredging must be addressed in order to avoid releasing risks for humans and the environment. French regulations come to govern the management of dredged sediments, considering them “safe” and possible to be dumped at sea or “contaminated” and needed to be treated on land as waste. In recent years, new constraints have been pushed toward the management of land. This management is, however, challenging as few channels are proposed to reuse marine sediments, and elimination appears to be economically and environmentally unsustainable. This study provides an overview of the technical and regulatory aspects related to dredged marine sediment management in France and aims to identify and discuss the limits of their valorization. Dredged sediments are mainly composed of particles with heterogeneous grain size, some being known for many applications such as building materials and growing media. However, several reasons have been put forward to explain why these particles are not reused when extracted from dredged sediments. Several technical, socio-economic, and regulatory obstacles explain the low demand for dredged sediments. This demand can be stimulated by government incentives and a good regulatory framework. National regulations could help streamline their reuse by removing their “waste” status and creating a regulated market for dredged sediment.

## Introduction

Sediments are materials formed by the union of particles of varying size or precipitated matter that have separately undergone some form of transport (Foucault et al. 2014). Some scientific disciplines consider that this transport takes place exclusively in the aqueous phase (Mathieu et Lozet 2011), but common sense also designates aeolian and glacial deposits as “sediment” (Minarro 2023). They are solid particles of sand, silt, clay, and other substances that result from the erosion of rocks, soils, and any continental solids and from the precipitation by biochemical processes in aquatic environments (Zonneveld et al. 2010). Natural and human activities are the vectors for the production and transport of sediments that settle at the bottom of water bodies, hindering navigation and impacting the physicochemical balance of these water bodies (Ferrans et al. 2019; Mehdizadeh et al. 2021; Pal and Hogland 2022). Consequently, regular dredging is necessary to maintain adequate depths in waterways (Chen et al. 2021). Dredging is the removal of sediment from the bottom of harbors, rivers, and other bodies of water. It is a very important operation for ports and waterborne transport systems in general, as it enables the construction of new waterways (Suedel et al. 2021) and their maintenance and secures the depth required for navigation (Baver 2015; Bianchini et al. 2019; Vogt et al. 2017). The anthropogenic activities throughout the globe generate large volumes of dredged sediments mainly in the harbors (Cox et al. 2021). Each year, 300 million m^3^ of sediments are dredged in Europe (Snellings et al. 2016) with around 50 million m^3^ in France (J. R. Harrington et al. 2016), against 100 million m^3^ in China and 300 million m^3^ in the USA (Florsheim et al. 2011). Whatever the reason for dredging, once removed from the water, sediments must be managed appropriately to minimize any potential negative impact on the environment.

Up to now, the most common management method for dredged marine sediments in the world is sea dumping, i.e., the immersion at sea of the extracted sedimentary material (Mousavi et al. 2023). The second main destination is onshore management, where they require prior treatment before eventually being reclaimed or stored in special containment facilities (Achour et al. 2014). Even if sea dumping remains the most widely used solution in terms of sediment volumes, controls on the nature of the materials to be dredged, their level of contamination, and impacts on dumping sites have been tightened (Abriak et al. 2023). Nowadays, the sea dumping option is increasingly criticized, and volumes are being reduced in favor of onshore management (Marmin et al. 2014). On land, national regulatory programs generally consider dredged sediments to be waste for disposal (Beddaa et al. 2020), potentially hazardous due to their high concentration of pollutants. Sediments classified as hazardous by current regulations must either be disposed of directly in hazardous waste storage facilities or treated to reduce their dangerous nature to acceptable levels. Given the large quantities of dredged sediment produced each year and the increasing regulatory requirements for sediment management, the conventional strategy of directly storing sediment in confined facilities is no longer sustainable from an economic, environmental, and social point of view (Barjoveanu et al. 2018; Mehdizadeh et al. 2021; Pal and Hogland 2022; Pellenz et al. 2020). Dredging and management of dredged sediments have even become a real challenge for port authorities (Loudini et al. 2020a, b; Loudini et al. 2020a, b). In this regard, many studies propose circular management of dredged sediments by reusing them as resources. Sediments remain, however, little reused today (Achour et al. 2019).

The appropriate recycling process for dredged sediments must be decided on the basis of their mechanical, chemical, and physical properties. For instance, the grain size of the dredged sediments must be considered to address the best management channel. Sand particles or fine-grained particle fractions could be used as a suitable raw material for many high-value and beneficial applications and processing options (Achour et al. 2019; Ayati et al. 2018; Balkaya 2018; Beddaa et al. 2020, 2022; Vincenzo et al. 2018; Martellotta et al. 2023; Soleimani et al. 2023a; Soleimani et al. 2023a, b, c; Zheng et al. 2019). The dredged sediment’s chemical quality is also of great importance in the definition of their becoming. Sediments can be contaminated from a variety of sources, whatever their location. Their recycling or reuse calls for a decontamination step. For the treatment of contaminated sediments, technological innovations have emerged. Among the different stabilization methods identified or proposed in the literature, the following techniques are noted: thermal treatment, chemical precipitation, biological treatment, carbonation, vitrification, stabilization/solidification (S/S) relying on the use of hydraulic binders (Elghali et al. 2022). The use of cementitious materials has proved to be effective in stabilizing a wide range of inorganic pollutants while remaining safe for the applicators and relatively low-cost (Wang et al. 2018a, b). Geotextile containers provide a reliable, cost-effective technology to collect, dewater, and permanently store contaminated sediment on or near the site (Bossy et al. 2023), which has been successfully used during dredged sediments dewatering treatment from Southern France (CCTP dredging Occitanie 2020), mine reclamation operations, and other environmental remediation projects worldwide (Lawson 2008).

However, current constraints seem to hamper the large-scale development of dredged sediment recycling. The recovery of resources from sediments has become a critical focus, aligning with the dual goals of environmental stewardship and resource sustainability (Liu et al. 2023; Mehmood et al. 2023). The utilization of sediment as a source of secondary materials presents a unique opportunity to progress toward a circular economy, transforming waste into valuable resources (Singh et al. 2023; Wang et al. 2019; Zhao et al. 2022). This paradigm shift requires a reimagining of sediments beyond their role as pollutant sinks, envisioning them instead as potential treasure troves of valuable materials (Capasso et al. 2021). However, it was observed that dredged sediments can act as a sink for emerging contaminants for which no remediation strategies are available (Singh et al. 2023). The field of sediment valorization, while nascent, is ripe for exploration, necessitating a holistic understanding of the technological, economic, and policy frameworks that govern resource recovery practices.

This study provides an overview of the technical and regulatory aspects associated with the management of dredged marine sediments. It will focus particularly on the French situation and aims to identify the actual reusing of dredged sediments as a resource and determine the potential limits of this reuse. The approach will mainly be made considering the specific situation of the South French Occitanie area, which has a significant maritime coastline (220 km) occupied by 5 fishing harbors, 70 marinas, and 3 commercial harbors. Beforehand, an overview of the main characteristics of dredged sediments, current legislation, and conventional sediment reclamation methods will be given. The limits and barriers to dredged sediments reused as a resource are addressed, and some recommendations on future policy options for the sustainable management of dredged sediments are finally proposed.

## Main characteristics and intrinsic qualities of sediments: constraints for their management

Before any discussion, it is important to underline some semantic and disciplinary details when talking about sediments. Several authors consider that sediments are made up of particles of varying size or precipitated matter that have undergone separate transport and that this transport takes place exclusively in the aqueous phase (Foucault et al. 2014; Mathieu et Lozet 2011). As a whole, sediment definitions are therefore based solely on the mode of formation of the object and not on its bio-physicochemical nature (with the exception of its “loose” character). As a result, both “pebbles” and the finest particles are part of the sediment (Minarro 2023). This peculiarity needs to be considered when it comes to legislating and making a method applicable at a national level. At present, for example, there are problems concerning the characterization of sediments prior to dredging in the presence of a majority coarse fraction, as is the case in mountain environments. Derived from soils and considered fine materials (almost all grains < 2 mm), the sampling methods and standards used to characterize such coarse samples are ultimately unsuitable. In the worst case, it is the very absence of a methodology to better characterize the stock of contaminants present in coarse sediments that biases the diagnosis and therefore limits opportunities for remediation and recovery.

Other categories are commonly used to designate sediments, but each has a connotation specific to the nature of the deposit. These include the terms “silt,” “silts,” or “mud.” For geologists, “mud” is a Dutch word that designates a “loose, waterlogged sediment with very fine detrital elements (sands, silts, clays), colloids largely of biological origin (bacteria, diatoms, pollens, humus, etc.), iron hydroxides and sulfides…” (Foucault et al. 2014).

Finally, for geologists and soil scientists alike, “mud” is a general term for any fine, waterlogged deposit that can flow easily (Foucault et al. 2014; Mathieu et Lozet 2011). Geologists also use it to designate deposits in ocean basins with high mineralogical clay content. However, the term “sludge” is widely used to designate various industrial processing residues (Mathieu et Lozet 2011). It is in this sense that the term “dredging sludge” is used in French regulations, particularly those relating to waste (e.g., Directive 1999/31/EC, Article R.541–8 of the French Environmental Code) (Légifrance 2012). On the contrary, the term “sediment” is used to designate these same materials when they are “displaced within surface waters,” a situation in which they are not considered waste. In addition, the term “dredging sludge” is used to distinguish between sediments dredged for maintenance and those dredged for civil engineering. In France, in 2011, aggregates of marine origin accounted for 2% of national production (Gautier et al. 2012). Finally, the terms “sludge” and “sediment” appear to be used with a certain degree of consistency in legislative documents; the term sludge is being connoted as “waste” in this context (and even though not all sludge is waste). However, it suggests that dredged sediments with waste status are necessarily fine, which is not always the case.

The main characteristics of dredged marine sediments vary greatly (Table 1). They vary from one region to another even if they are close. Sediments have a varying nature but a functioning controlled by a limited number of parameters. There are many factors that can explain these differences on the nature of the sediment (Safhi et al. 2020):

- the mineralogical nature and size of deposited particles;
- the geochemical and hydrodynamic conditions of the deposition environment;
- the degree of erosion, inputs of organic matter from the watershed;
- the climate (temperature);
- the biological activity of the medium;
- the economic activities in the surrounding area, or even the dredging of sediments.

**Table 1:** Main characteristics of dredged sediments from different locations

		Reference									
		Tessier et al. (2011)	Dorleon et al. (2024)	Bel Hadj et al. (2014)	Doni et al. (2018)	Ferrans et al. (2021)	Lopez et al. (2022)	Norén et al. (2020)	Todaro et al. (2018)	Zhao et al. (2022)	Zhou et al. (2022)
		Harbor, location									
		Toulon, France	10 harbors, South of France	Rades, Tunisia	Livorno, Italy	Malmfjärden, Sweden	Galveston, USA	Gothenburg, Sweden	Taranto, Italy	Tai Lake, China	Lamma, Hong Kong
Granulometry %	Gravel	-	11.2	< 1	N.D	–	–	–	–	–	48.7
	Sand		31.16	≈ 18	63	10–20	–	–	19.4	–	51.1
	Silt–clay	70	57.64	≈ 81	37	80–90	–	–	80.6	–	0.2
Physico-chemical properties	Humidity %	-	35.2	65–80	–	70–78	–	–	44.8	89.9	23.9
	pH	8.1	8.35	–	8.3	6.7	–	–	8.8	–	8.1
	Conductivity µS·cm^−1^	-	–	–	16,8	–	–	–	4,1	–	–
Nutrients mg·kg−1	TN	-	1.7	–	3,84	8563–9488	–	–	–	–	–
	NH_4_	-	–	–	14.1	–	–	–	–	–	–
	NO_3_	-	–	–	20.0	–	–	–	–	–	–
	TP	-	833.8	–	650	740–1159	–	–	–	–	–
Organic compounds mg·kg^−1^	TOC	0.52–13.244	37.68	67,600–68,300	19,3	129	–	–	123	5741	25,5
	TBT µg·kg^−1^		170	–	–	–	–	150	–	–	–
	PAHs µg·kg^−1^		1307	–	–	–	–	–	5,389	–	–
	PCBs µg·kg^−1^		52.4	–	–	–	–	–	1,669	–	–
Metals mg·kg^−1^	Cu	5.8–846	91.88	–	123	40	0.8–351.8	50	10.5	70.85	378.5
	Zn	24.3–1340	101.48	–	240	120	4.3–336.6	200	16.6	104.3	138.5
	Cd	0.042	0.21	–	N.D	N.D	0.02–0.5	0.4	–	–	1.8
	Cr	8.5–121	27.35	–	64.0	24	1.5–70.8	40	54.0	84.49	29.7
	Ni	8.8–37.6	18.01	–	49.1	23	1.1–30.3	20	38.2	–	23.6
	Pb	14.9–469	28.64	–	59.7	44.9	1.8–29.2	40	87.4	65.06	101.1
	As	6.7–61	10.66	–	–	8.3	1.3–20.0	–	12.3	–	7.8
	Co	–	–	–	–	–	–	–	7.1	–	–
	Al	4860–59.273	17,111.11	–	–	–	1,6–54,003	–	–	–	–
	Fe	–	–	–	–	–	636–23,655	–	–	–	–
	Sb	–	–	–	–	–	0.1–1.5	–	–	–	–
	Mn	–	–	–	–	–	6.1–694.5	–	–	–	–
	Hg	–	0.13	–	–	–	0.01–0.4	–	–	–	–

*N.D.* non-detected, *NH*_*4*_ ammonium, *NO*_*3*_ nitrate, *PAHs* polycyclic aromatic hydrocarbons, *PCBs* polychlorinated biphenyls, *TOC* total organic carbon, *TBT* tributyltin, *TN* total nitrogen, *TP* total -phosphorus

These factors are interdependent. For example, the size of deposited particles depends in part on the hydrodynamic conditions of the deposition medium. The chemical conditions of the environment are themselves highly dependent on the exchange of dissolved oxygen between the sediment and the water column, and therefore on the permeability of the sediment (a function of particle size and spatial arrangement), but also on biological activity, itself, dependent on the presence of organic matter and temperature. Therefore, initial sediment characterization is essential to determine what management strategy is necessary.

The minerals most commonly identified in dredged sediments are quartz, feldspars, calcite, and alumino-silicates (illite, kaolinite, etc.) (Aouad et al. 2012; Brakni et al. 2007; Kribi et al. 2012). Other minerals are also sometimes identified, but in lesser proportions: dolomite, halite, muscovite, pyrite, etc. Silicates and carbonates are used in the composition of cement clinkers. According to Miraoui et al. (2012), the chemical composition by weight of oxide is similar in sediments from the same port: silica (SiO_2_) 47%, lime (CaO) 15%, and alumina (Al_2_O_3_) 14%. Similar proportions were measured by Ben Allal et al. (2011) on sediments from two Moroccan ports. According to these authors, the composition of these sediments is comparable to that of clays used in the production of clay bricks. Sediment minerals are either inherited from the watershed (quartz, feldspar, muscovite, smectite, calcite, hematite, and other iron hydroxides, etc.), or newly formed following transformations of inherited minerals, or precipitation of new compounds. For the former, it is the iron-containing minerals that will undergo the most significant transformations if the environment is low in oxygen (Bataillard et al. 2014). The latter are generally carbonates and sulfides, whose formation kinetics are controlled by biological activity, linked to their metabolism of organic matter degradation, and the availability of oxygen. As iron hydroxides and organic matter are ubiquitous compounds in the environment, the same chemical reactions are expected in all sediments. In the end, oxygen availability is the most important criterion for understanding sediment chemistry.

If oxygen (O_2_) diffused as quickly in water as in air and was not consumed by biota in the water column, sediments would not have a different chemistry to soils. Molecular diffusion in water is some 10,000 times slower than in air (Fiessinger 1970). Oxygen is the main oxidizing agent for living organisms, which use it as an electron acceptor in oxidation–reduction reactions to obtain the energy they need for their development. Oxygen consumption is high, and diffusion in water generally does not provide sufficient O_2_ in sediments, which are the site of intense biological activity. The other way of bringing O_2_ into the sediment is through convection, i.e., the circulation of water containing dissolved O_2_. This is what happens in environments combining strong currents and permeable sediments, such as mountain rivers with coarse sedimentation, and coastal areas subject to tides or currents, with sediments mainly made up of sand and gravel. These are the types of sediments that can be mined. This part of the sediment is strongly influenced by the convection of river water and is known as the “hyporheic zone” (Liu et al. 2017).

In calmer zones with less permeable sediments, convective phenomena are limited, and the environment can rapidly run out of oxygen. Under these conditions, bacteria adapt and use other electron acceptors for their metabolism (Otaño-Cruz et al. 2017). Redox equilibrium involves two chemical couples, consisting of a reduced form and a conjugated oxidized form. Consequently, in sediments, a succession of equilibria is established involving a series of electron acceptors other than oxygen (Sigg 2000). In recently deposited sediments, nitrate (NO_3_^−^) is successively reduced, followed by manganese (Mn(III and IV)), iron (Fe(III)), sulfate (SO_4_^2−^), and finally carbon dioxide (CO_2_). The gradual disappearance of brown iron hydroxides, the appearance of more or less well-crystallized sulfide minerals, often black in color, and the presence of organic matter, also black, explain the differences in hue often observed in sediments (Sigg 2000). Redox potential is therefore a key parameter in understanding how sediments function. But its measurement is tricky, as it requires the sample to be protected from contact with air. As a first approach, knowledge of the deposition context and simple measurement of the parameters “granulometry” and “organic and inorganic carbon content” may suffice to give an initial idea of the processes governing the current functioning of the sediment.

The nature of sediments often offers favorable conditions for trapping metals and hydrophobic organic pollutants. The redox couple (SO_4_^2−^/HS^−^) plays a major role in the retention of mineral contaminants (Zn, Cd, Pb, Hg, etc.) in sediments, as many trace elements form highly insoluble sulfide precipitates. Numerous studies show that sulfate reduction (reduction of sulfate anions to sulfides) commonly occurs in river and harbor sediments and plays a role in trapping metal contaminants (Boyd 1995; Morse and Luther 1999; Tack and Verloo 1995).

Furthermore, anaerobic respiration does not offer the same energy yields as oxygenated environments. Microbial degradation of organic compounds is often incomplete, leading to their accumulation in the sediment and the maintenance of reducing conditions in the absence of disturbed equilibria. Generally speaking, it is not uncommon to observe organic matter levels of the order of 5 to 10% in dredged sediments from low-flow rivers and lakes. Natural organic molecules have a large specific surface area and a variety of chemical groupings, making them a highly reactive compartment. Pollutants may be directly associated with them through organo-mineral complexation or, more generally, because they have a particular affinity for this compartment (as in the case of hydrophobic organic molecules).

These phenomena explain why sediments are considered pollutant sinks. Pollutants are generally tightly bound to the solid matrix, which itself changes little if reducing conditions persist. These trapping reactions are reversible: dredging and deposition on land of sediments in contact with the atmosphere induce oxidative dissolution of sulfides and mineralization of organic matter. Numerous studies have shown that this return to aerobic conditions is likely to lead to greater mobility of certain metals and greater toxicity of leachates (Li et al. 2023; Panfili et al. 2005; Piou et al. 2009). This phenomenon needs to be considered in land-based sediment management projects, particularly in the case of prolonged storage prior to reclamation.

## European and specific French regulatory framework for the management of dredged sediments

There are a few directives, laws, and regulations that directly or indirectly address sediment management at the European level, dealing with different legal and technical backgrounds and purposes. Guidelines that are of high relevance for sediment management exist mostly for the purpose of environmentally sound handling for navigation. For the maintenance of waterways and harbors, sediments have to be handled in an environmentally sound and economical way. The way to find the appropriate regulation within legislation is to ask the question: What will happen with the dredged material/sediment?

When dredged sediments have to be relocated or disposed within the water (e.g., in a confined or contained way), European water legislation has to be considered. The water legislation is being significantly changed by the Water Framework Directive (WFD 2000/60/EC of October 23, 2000; 2013/39/EU of August 24, 2013). Despite the fact that the terms “sediment” and “dredged material” are rarely mentioned in this WFD, sediments are a natural and essential part of the aquatic environment, and their management has an important role within water legislation. It is obvious that a comprehensive sediment management concept has to be part of each river basin management plan (RBMP), which has to be produced and updated by each EU member country to fulfill the demands of WFD.

When dredged sediments have to be managed on land, the waste legislation has to be considered regarding mainly the first article of the European Waste Directive (75/442/EEC) according to which a dredged sediment can be defined as a waste: “waste means any substance or object which the holder disposes of or is required to dispose of pursuant to the provisions of national law in force.” This definition is independent from the quality of the sediment. The European Waste Catalogue (2001) contains two waste codes for sediment (170505 “Dredging spoil containing dangerous substances” and 170506 “Dredging spoil other than those mentioned in 170505”). If sediments are highly contaminated, they have to be classified as hazardous waste (waste code 170505 in the European Waste Catalogue), and the Basel Convention on the control of trans-boundary movement of hazardous waste and their disposal (1989/1990) has to be considered when the sediments are transported (after dredging) into other countries for the purpose of treatment and disposal. A waste is considered hazardous if it has any of the fifteen hazard properties listed in Annex III of Directive 2008/98/CE of November 19, 2008, on waste (EEC 2008). The handling of waste legislation follows the principles (1) avoidance of waste, (2) beneficial use (including treatment), and (3) landfill. All three options are part of an integrated sediment management, and there are several technical guidelines in waste legislation which apply for sediments and differ, to some extent, on the national level. Uncertainties with the handling of dredged sediments in the case of treatment and land filling are expressed in the preamble of the European Landfill Directive (1999): “Whereas further consideration should be given to the … processing of dredging sludges.”

It must be underlined that when dredged sediments remain environmentally sound in the aquatic environment, the term “waste” is not to be used because water or waterway legislation applies in this case. This is expressed in the exception of the European Landfill Directive in Article 3 that “… the deposit of non-hazardous dredging sludges … in surface water including the bed and its sub-soil is exempted from this directive.” Aquatic disposal can be practiced in an environmentally sound way for the overwhelming amount of dredged sediments worldwide. Sediments are in the first case natural and important part of the aquatic system and cannot be taken out unrestrictedly without negative consequences for the water system. A purely formal classification as waste would cause a negative image (which is already there) and unnecessary troubles in permitting procedures for aquatic disposal, which may lead to an inadequate handling. European countries are dealing in different ways with this problem currently.

In the framework of these European legislations, a specific consideration of the French situation can be made. The French reglementation was introduced by the decree of 14 June 2000 (Légifrance 2000). The official circular N°2000–62 completed this decree by offering a reading grid. The objective is to assess the impact that a planned operation may have, particularly on the aquatic environment through levels of contamination known as N1 (high quality) and N2 (medium quality). These N1 and N2 threshold values will therefore target contaminants known to have harmful effects on the environment, both TMEs (trace element metal) and organic molecules. They, however, do not yet consider emerging contaminants (Mo et al. 2019; Pintado-Herrera et al. 2017). The N1 and N2 levels are therefore benchmarks for deciding on the administrative regime of a dredging operation (declaration or authorization) and for orienting an operation either toward the dumping of sediments or toward their management on land. It should be noted that in this summary, we are only interested in the field of marine or estuarine sediments, which includes harbor sediments. In order to evaluate the effect of a disposal operation and its environmental quality, an interministerial order was established on August 9, 2006 (AIDA 2006). According to this decree, the threshold levels N1/N2 (Table 2) are defined as follows: (i) below level N1: potential negligible impact, sea disposal possible without any further investigation; (ii) between levels N1 and N2: further investigation may be necessary depending on the project considered and the degree to which level N1 is exceeded; and (iii) above level N2: potential risk of negative impact of sea disposal, need for further investigation. Several amending orders (AIDA 2014, 2020) have led to changes in the threshold values initially defined in the order of 9 August 2006. The levels in force in 2022 are shown in Table 2.

**Table 2:** Current N1 and N2 levels for sea dumping of marine and estuarine sediments (AIDA 2020)

Substance	Level N1	Level N2
**Trace elements (in mg.kg**^**−1**^** dry matter)**		
As	25	50
Cd	1.2	2.4
Cr	90	180
Cu	45	90
Hg	0.4	0.8
Ni	37	74
Pb	100	200
Zn	276	552
**Polychlorinated biphenyls—PCB (in µg.kg**^**−1**^** dry matter)**		
Congener 28	5	10
Congener52	5	10
Congener 101	10	20
Congener 118	10	20
Congener 138	20	40
Congener 153	20	40
Congener 180	10	20
**Polycyclic aromatic hydrocarbons—PAH (in µg.kg**^**−1**^** dry matter)**		
Naphthalene	160	1130
Acenaphthene	15	260
Acenaphthylene	40	340
Fluorene	20	280
Anthracene	85	590
Phenanthrene	240	870
Fluoranthene	600	2850
Pyrene	500	1500
Benzo [a] anthracene	260	930
Chrysene	380	1590
Benzo [b] fluoranthene	400	900
Benzo [k] fluoranthene	200	400
Benzo [a] pyrene	430	1015
Di benzo [a, h] anthracene	60	160
Benzo [g, h, j] perylene	1700	5650
Indeno [1,2,3 -cd] pyrene	1700	5650
**Tributyltin—TBT (in µg.kg**^**−1**^** dry matter)**		
TBT	100	400

N1 (low pollutant concentrations), N2 (mid pollutant concentrations)

The purpose of these reference levels (N1/N2 for marine and estuarine sediments) is to set markers to anticipate the environmental impact of a planned sediment material handling operation, particularly dredging (Keddari et al. 2020). Consequently, these threshold values also tend to guide the choice in the management mode of sediments with (i) either a return to the aquatic environment by dumping at sea and/or (ii) a management on land if the sediments do not present an adequate physicochemical and environmental quality or if the local conditions do not allow a return to the aquatic environment. In practice, when the sediment analyses do not show that the N1 level is exceeded or is between N1 and N2 with strict reservations, dumping is generally the solution chosen, assuming that the transport methods at the sea are technically and economically acceptable. These last criteria can also be a determining parameter in the choice of the management solution. Above the N2 threshold, onshore management is preferred if it is not environmentally damaging (Achour et al. 2014).

## Dumping at the sea: regulation and environmental issues

**Fig. 1:**
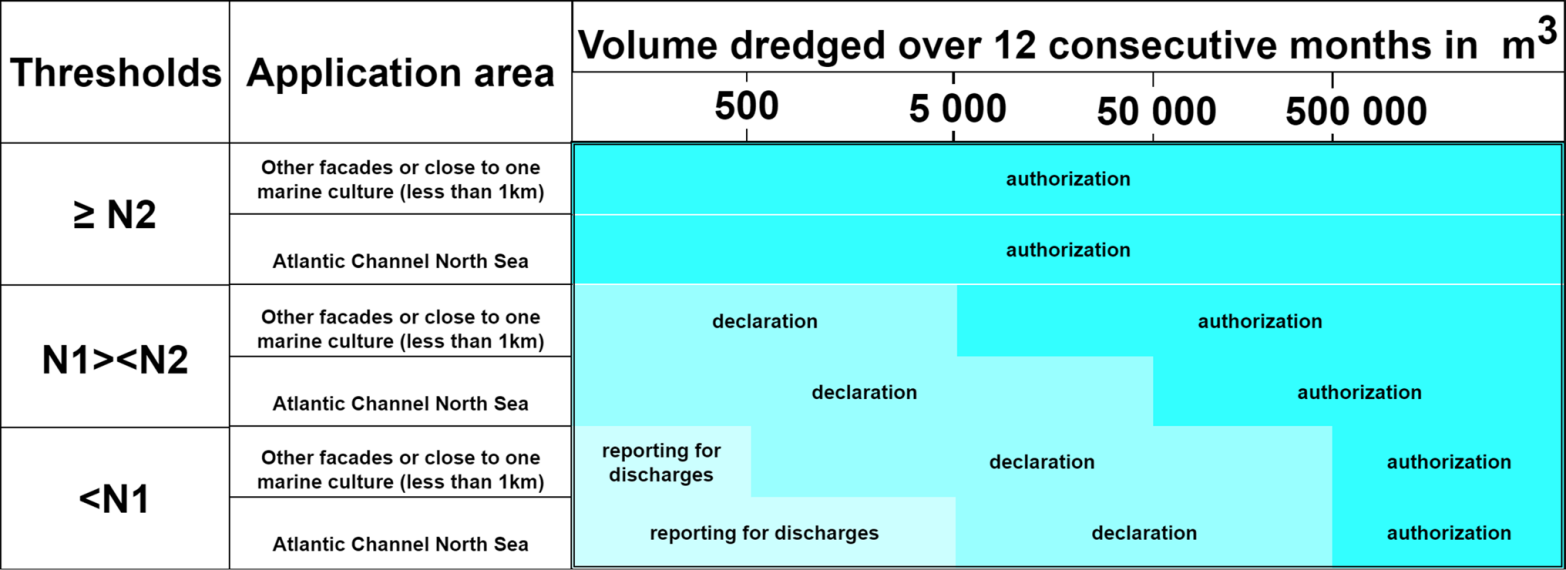
Application of the water law (Article R214-1 to 6 of the Environmental Code) (Légifrance 2012). To know the procedure applicable to an operation, it is necessary to know the volume to be dredged in a period of 12 months, the content of the sediments in relation to the reference levels for sea dumping of marine and estuarine sediments (N1: low pollutant concentrations, N2: mid pollutant concentrations), and the distance of the discharge compared to a shellfish or marine culture area, according to the Cerema technical note (2021). N1, N2: reference level defined by the French Order of August 9, 2006

In France, dumping of sediments at the sea is regulated by Law no. 76–599 of July 7, 1976, relating to the prevention and repression of marine pollution and to the fight against accidental marine pollution, and by Decree no. 82–842 of September 29, 1982, taken for the application of the aforementioned law (Robin 1986). This decree was repealed by decree no. 2006–880 of 17 July 2006 (AIDA 2006), relating to the authorization and declaration procedures provided for by articles L 214–1 to L 214–3 of the Environment Code for the protection of water and aquatic environments and which defines all measures relating to dumping permits (Légifrance 2012) (Fig. 1). Furthermore, when the project of dumping at sea is likely to significantly affect a Natura 2000 site within the meaning of Article L. 414–4, the “water law” impact document must include an assessment of its impact with regard to the conservation objectives of the site. In other cases, dumping techniques are neither possible nor desirable given the various environmental (absence of current in the canals or clogging of the bottom, for example) or sanitary imperatives that must be considered (protection of zones designated for the protection of economically important aquatic species) and land-based management must then be considered.

Insofar as a “permit” is required in the case of dumping in application of international conventions, all maritime dredging operations giving rise to dumping are therefore, at the very least, subject to declaration as specified in heading 4.1.3.0 of the nomenclature annexed to article R. 214–1 of the Environmental Code (Fig. 1). The “water law” declaration is consistent with the concept of “permit” insofar as this same ordinance introduces the possibility for the prefect to impose specific requirements on a declaration, or even to oppose it. In contrast, these regulatory levels are useful for positioning the sediments to be dredged according to their physicochemical quality. In addition, these reference levels are intended, in combination with the volumes dredged, to define the administrative regime applicable to the dredging operation. Depending on the position of the sediments in relation to the thresholds and the volumes to be dredged, the extraction operation may fall under the declaration or authorization regime (Fig. 1). Dredging and dumping operations are subject to either declaration or authorization, depending on N1 and N2 contamination levels, the volumes involved, the distance from shellfish farming areas, and the seafront concerned.

**Fig. 2:**
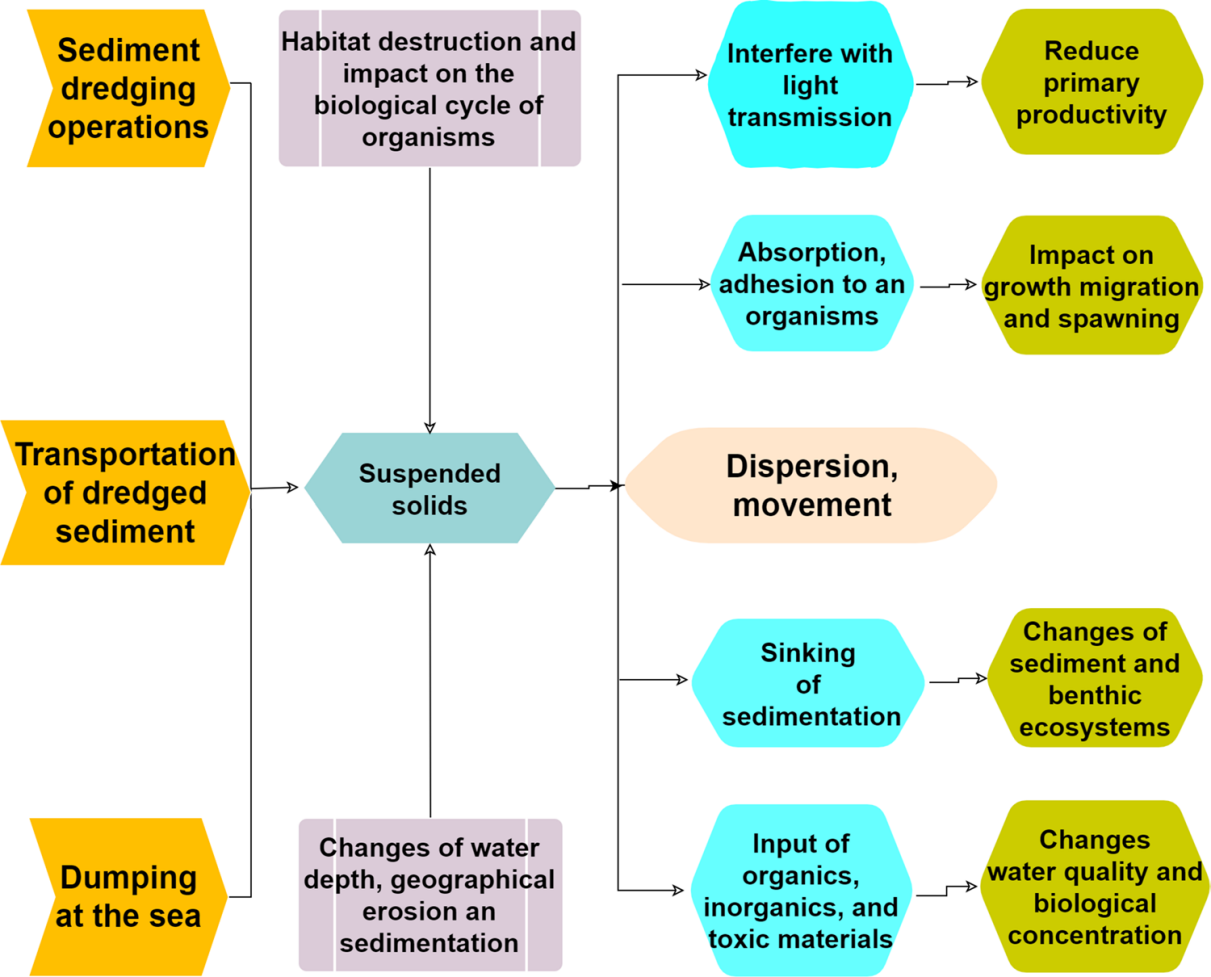
Environmental issues of dredging and sea dumping of dredged sediment on marine ecosystems

Dumping and resuspension operations concern more than 90% of the material dredged in French ports (J. R. Harrington et al. 2016). However, the dumping of sediments at sea is often limited by technical difficulties (access to dredging areas by lapping barges), the location of dumping sites, which are sometimes too far from the port, and economic costs that are too high for port managers (Beddaa et al. 2020). The dumping of dredging sediments in the ocean can induce several environmental issues and have an adverse impact on the marine environment and its ecosystems (Fig. 2) by causing changes in water depths, bottom morphology, and current velocity. Undesirable consequences include erosion and sedimentation, the destruction of habitats, and impact on water quality such as increased turbidity. Both the pelagic and benthic ecosystems can be affected as suspended solids spread and sink (Licursi et al. 2009; Jeuken et al. 2010; Simonini et al. 2005; Stronkhorst et al. 2003; Wolanski et al. 1992; Zimmerman et al. 2003). In particular, there is a strong possibility that toxic and hazardous matter in dredged sediment can accumulate within fishery resources.

## Return to the land: analyses inherent to the status of waste for dredged sediments managed on land

When dredged sediments cannot be dumped, they must be managed on land. In the absence of specific regulations, the management of these sediments, now considered as waste, is governed by the legislation on Installations Classified for the Protection of the Environment (ICPE), which are controlled by the Regional Directorates for the Environment, Development and Housing (DREAL) in France (AIDA 2020). The main objective of these institutions is to promote sustainable development and ecological transition. Regarding the dredging of sediment issue, they, therefore, work on a regional scale in partnership with local institutions involved in dredging operations and sediment management in order to develop regional guides, reference systems, management plans, and recovery of dredged materials and thus allow for a common line of action at the level of the various stakeholders. When the immersion of sediments is not possible or not desirable, two management methods can be envisaged, namely disposal or, in the case of non-hazardous sediments, valorization.

**Fig. 3:**
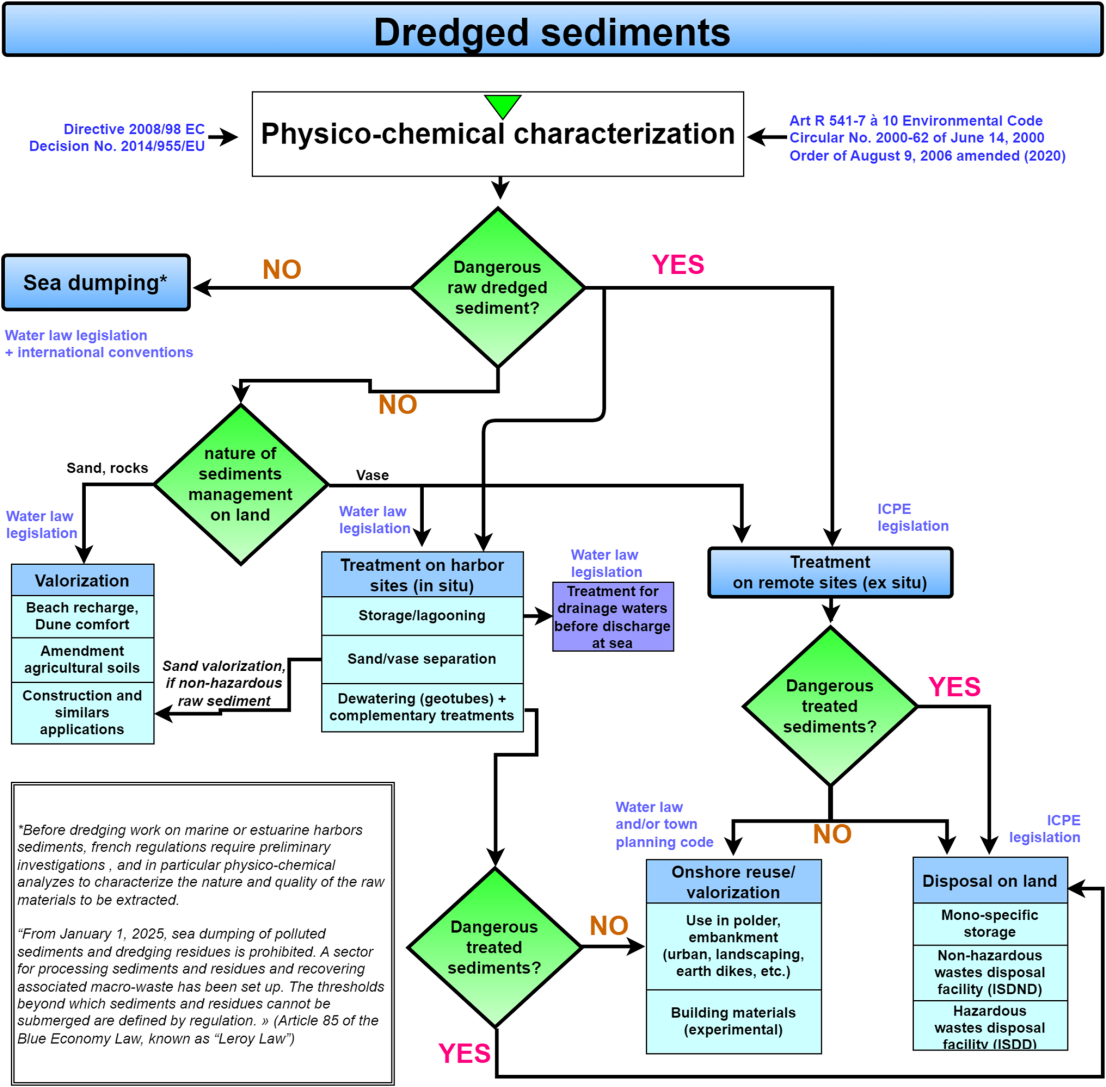
Overview of the actual French regulatory framework for the management of dredged sediments according to the Environmental Code, Annex I of Article L541-8, the Order of February 15, 2016, and the Order of June 30, 2020

In the case of disposal, sediments could be either sent to incineration centers, an economically disadvantageous and unsustainable solution, or are redirected to waste disposal facilities. These facilities are classified in three categories in France considering the waste chemical quality (Table 2): (i) Inert Waste Disposal Facilities (ISDI), whose acceptability criteria are defined in the decree of October 28, 2010, on inert waste (Légifrance 2010); (ii) Non-Hazardous Waste Disposal Facilities (ISDND), those are regulated by the decrees of September 9, 1997, December 12, 2014, and February 15, 2016 (AIDA 2014, 2016; Légifrance 1997); and (iii) Hazardous Waste Disposal Facilities (ISDD) subjected to the decree of December 30, 2002 (Légifrance 2002a). In case the thresholds presented in Table 2 are exceeded, treatments must be applied to the wastes/sediments prior to storage as highlighted in the decree of December 12, 2014, and February 15, 2016 (AIDA 2014, 2016). The (obligatory) characterization of sediments managed on land allows them to be directed to the appropriate management channels with regard to ICPE regulations (Balet, 2016). As such, the sediments must be characterized with regard to the waste reference (Légifrance 2002b, 2014), which includes (i) on the one hand, the determination of their hazardous or non-hazardous character and (ii) on the other hand, the analysis of their inert or non-inert character. In practice, it is first determined whether the sediment is inert, and then their dangerous nature will be evaluated if this first test reveals the contrary (Fig. 3).

Regarding the assessment of the inert character of sediments (Fig. 3), it is carried out on the basis of the parameters contained in the orders of 12 December 2014 (repealed on 15 February 2016), relating to the conditions of admission of inert waste in facilities falling under headings 2515, 2516, 2517, and in inert waste storage facilities falling under heading 2760 of the nomenclature of classified facilities for the Protection of the Environment (ICPE) (AIDA 2016; Légifrance 2014). The methodologies and thresholds used to assess the inert nature of sediments (Table 3) concern all wastes and therefore do not consider the specifics of dredged material. Thus, the origin and the nature of these materials can be penalizing and lead to the exceeding of certain thresholds. For instance, the maximum leaching values for chlorides, sulfates, and soluble fractions can be exceeded by sediments of marine origin without these materials being contaminated by pollutants of anthropic origin.

**Table 3:** Limit values for waste acceptance in Inert Waste Disposal Facility (ISDI), Non-Hazardous Waste Disposal Facility (ISDND), Hazardous Waste Disposal Facility (ISDD) of the Decree of December 12, 2014 (results obtained after leachate test). Waste thresholds: ISDI, ISDND, ISDD (Order of December 12, 2014). Thresholds Sediment waste: ISDS (Order of February 15, 2016; Jun 30, 2020)

Parameters	ISDI	ISDND or ISDS	ISDD or ISDSD
	Analyses on leachate: values expressed in mg/kg of dry matter according to standard test NF EN 12547–2		
Arsenic (As)	0.5	2	25
Barium (Ba)	20	100	300
Cadmium (Cd)	0.04	1	5
Chromium (Cr)	0.5	10	70
Copper (Cu)	2	50	100
Mercury (Hg)	0.01	0.2	2
Molybdenum (Mo)	0.5	10	30
Nickel (Ni)	0.4	10	40
Lead (Pb)	0.5	10	50
Antimony (Sb)	0.06	0.7	5
Selenium (Se)	0.1	0.5	7
Zinc (Zn)	4	50	200
Chloride (Cl^−^)	800	15,000	25,000
Fluoride (F^−^)	10	150	500
Sulfates (SO_4_^2−^)	1000	20,000	50,000
Soluble fraction	4000	60,000	100,000
**TOC**	500	800	1000

*TOC* total organic carbon

To assess the dangerous nature of sediments, it is necessary to refer to the nomenclature on waste (Annex II of Article R.541–8 repealed by Decree No. 2016–288 of March 10, 2016) (Légifrance 2016). According to this text, wastes have two entries, called “mirror entries”: 17 05 05 and 17 05 05*. The presence or absence of the asterisk respectively accounts for the hazardous or non-hazardous nature of the dredged material. In addition, sediments may have a reinforced natural radioactivity or show readings of radioactivity after exposure to an artificial source. Thus, it is advisable to follow the requirements of the ministerial order of February 15, 2016, on hazardous waste storage facilities. It is possible to refer to the circular of July 30, 2003 (AIDA 2003), relating to the procedures to be followed in the event of triggering of a radioactivity detection portal on storage facilities, incineration facilities, scrap metal recovery sites, and foundries, and to the ANDRA guide for the removal of radioactive waste (ANDRA 2009).

## Potential application for the beneficial reuse of dredged sediments

Unmanaged dumping of dredged sediments leads to damage to environmental and ecological systems. On the other hand, managing the land of these materials considered waste became a crucial challenge (Bortali et al. 2023; Miraoui et al. 2012; Slimanou et al. 2020; Wang et al. 2018a, b). For instance, due to a shortage of land, the cost of waste disposal gradually increases.

Using dredged sediments through a recycling process appears to be a sustainable approach toward supplementing natural resources, reducing pollution and greenhouse gas, energy optimization, enhancing economic development (Soleimani et al. 2023a; Soleimani et al. 2023a, b, c), improving sustainability on marine ecosystems. Among the potential application for the beneficial reuse of dredged sediments (Table 4), the following have been selected:

- Dredged sediments as aggregates for concrete and similar applications
- Dredged sediments as clinkers for cement and similar applications
- Dredged sediments for the manufacture of bricks and expanded aggregates
- Dredged sediments as plant growth medium

**Table 4:** Overview of reviewed studies on the beneficial reuse of dredged sediments (DS). Today, it is not clear if the beneficial reuse of dredged sediment as raw material for cement production is economically acceptable for full-sale applications

	Location/harbor								
	Dunkirk, France	Brittany, France	Napoli, Italy	Dunkirk, France	Dunkirk, France	Dunkirk, France	Dunkirk, France	French ports of the English channel	Napoli, Italy
Replacement description	0–30% binder	8%, 16%, and 33% of CEM I (52.5)		ROLAC®645 binder (6–8%)	Replacing up to 60 fine sediments with dredged material	6% OPC or blended cement with limestone and slag	-		Non-structural cemented mortar
Supplementary material	Cement, lime, class-F fly ash	Limestone	Fly ash, HNO_3_, HCl, HF, H_3_BO_3_	Hydraulic binder ROLAC®645, fly ash	Cement and quicklime	Limestone, slag, lime	20 and 80% phosphoric acid	Quicklime, CEMII 32.5	Blast furnace slag, ordinary Portland cement
Optimum result	Dredged soil mix with 9% cement	8% replacement with heating at 650 °C	10% fly ash replaced by dredged material	With an increase in binder content strength also increases	was obtained when adding both lime and cement	Max dry unit wt. 2.04 g/cm^3^; optimum water content is 11.6%	Both acids gave the same results	3% of quicklime and 6% of cement CEMII 32.5	Processed sediments with 80 µm size, 80% replacement of slag with standard Portland cement
Treatment	-	Treated at high temperatures (650 and 850 °C) to eliminate all organic compounds and activate the clay minerals; washing to remove chloride content	Calcination at 550 °C for 2 h	Natural dewatering, sieving	Decantation	Dewatering, lime addition	Novosol® process, calcination	Grinding (under 2 mm), dehydration in the oven at 40 °C, sediments crushing	Bioremediation, stored for 5 years in darkness at 4 °C, dried in a furnace at 45 °C
Outcome	Class-F fly ash is incapable of improving the resistance to thawing-freezing and water immersion	Hydration process required more time to complete; apparent porosity increased; at 33%, blended cement permeability decreased; strength decreased but within limits	Reducing emissions by 80% compared to Portland cement	Dredged material stabilized by a chemical binder can be used for subbase or base course material	Addition of lime with cement can change mechanical classification after 360 days	Salinity of the sediments is equal to 31.4 g/L; 4.5% of organic matter	100% substitution after treatment	Sediments are fine materials with high organic matter and clay activity	Use of dredged sediments in combination with fly ash can lead to geopolymeric matrices with interesting mechanical performances
References	R. Liu et Coffman (2016)	Liu et al. (2018)	Ramaroson et al. (2009)	Hamer and Karius (2002)	D. Wang, Zentar, et Abriak (2018)	J. Liu et al. (2012)	Dia et al. (2014)	D. Colin (2008)	Lirer et al. (2017)

Given the challenges associated with the current approach to sediment management in southern France, many stakeholders have expressed a desire to make the sediment system more resilient and sustainable. The approaches discussed include both the reuse of dredged materials to nourish beaches or as aggregates for concrete and other promising applications in the civil or environmental engineering field (clinkers for cement and similar applications, manufacturing of bricks and expanded aggregates, medium of plant growth). We have chosen 4 specific applications with regard to current policy at the local level (Occitanie, southern France) encouraging the search for local, environmentally friendly solutions for the reuse of sediments dredged from harbors of the Occitanie region (CCTP dredging Occitanie 2020). These approaches are not only seen as enabling adaptation to changes in development patterns but also to potentially reduce dredging costs.

### Dredged sediments as aggregates for concrete and similar applications

This application concerns either the sandy fraction of the sediment obtained after particle size separation, or the fine sediment as a whole, which is then used as filling or filtering material (Faure et al. 2019). Even if applicable, studies have shown that high salinity reduces the mechanical strength of cements made from marine sediments (Kaushik and Islam 1995). For this reason, Siham et al. (2008) use lime to reduce the effect of sodium, increase the dryness of dredged sediments, and stabilize organic matter. Further additions of sand and then Portland cement are made to obtain the qualities required for intensive road use (base layer on which the bitumen rests, and sub-base layer on which the base layer rests). An example, the final product contains 27% dredged fine sediments (grain size < 63 µm at 90%), 67% sand, and 6% cement (dry equivalent) (Bortali et al. 2023; De Gisi et al. 2020; Gupta et al. 2023; Todaro et al. 2023; Wang et al. 2018a, b). Still, in the case of road material formulations, mixing tests have shown that sediment incorporation reduces material elasticity linearly with the incorporated content (Agostini et al. 2007; Han and Wang 2016). However, compressive and tensile strengths are initially improved (by a few percentage points) for additions of between 5 and 20% of treated sediment, then fall beyond that (Han and Wang 2016). These results may be explained by the low aggregate strength of treated materials. The advantages of mixtures in limited proportions would be due to the filling effect of the added fines (increased skeletal compactness), optimization of cement hydration (more nucleation sites thanks to the presence of fines), and better mechanical compatibility between aggregates and paste, reducing the risk of cracking during setting and drying. Hence, the organic matter present in dredged sediments can retard the setting of cements and reduce their mechanical performance (Maletić et al. 2019; Mehdizadeh et al. 2021; Zhao et al. 2022). The manufacture of concrete from sediment is therefore conceivable, but for non-structural applications. It would essentially be used as an underlay for traffic lanes (Ferrans et al. 2019).

### Dredged sediments as clinkers for cement and similar applications

The use of sediments not as aggregate but as raw material for clinker production has also been the subject of researches (Aouad et al. 2012; Kou et al. 2021; Wang et al. 2019; Y. Zhou et al. 2022). Generally made up of marls (loose rocks made up of limestone and clay), clinker is the result of calcination at 1450 °C of the rock. The latter must contain approximately 75% limestone and 25% clay. Reduced to powder and then mixed with gypsum and various other additives, clinker constitutes Portland cement. The anhydrous oxides of the clinker will cause the cement to solidify through hydration and carbonation.

Aouad et al. (2012) show in the laboratory that a sediment from the canals of Northern France, corrected by various additions, makes it possible to obtain a cement with qualities comparable to those of commercial formulations. The final rate of incorporated sediment is 39% of the total mass of the materials, calculated on the basis of the characteristics of the latter and the different indices necessary for the formulation of the clinker. Very specific additions such as pure alumina (Al_2_O_3_), hematite (Fe_2_O_3_), and calcium carbonate have, for example, been made.

Dalton et al. (2004) also obtained very satisfactory results regarding the quality of Portland cements made from clinker containing between 1 and 12% by mass of marine sediments from the port of New York (USA). However, these authors believe that a higher calcination temperature is necessary to transform the quartz in the sediment into amorphous silica. However, the chlorine contained in marine sediments can have a strong impact on the quality of the clinker. Zhao et al. (2018) show that the presence of this element (and sulfite) during the rise in temperature eliminates alkaline cations by volatilization, thus reducing the quantity of active lime (CaO) and correlatively the CaO/SiO_2_ ratio. Cl and SO_3_ are also incorporated into the structure of the clinker minerals, which reduces their reactivity (and therefore their hydration capacity). This leads to a low-strength cement. Volatilized alkaline chlorides (CaCl_2_, NaCl, and KCl) precipitate in the smoke evacuation pipes of manufacturing plants, which increases maintenance costs. Finally, chlorides can cause corrosion of concrete reinforcement steels. Dalton et al. (2004) estimate that these disadvantages can be overcome (Cl effect and higher heating T°) if the incorporation of sediment does not exceed a few percents of the total (between 3 and 6%). However, they recognize an increase in the maintenance costs of the installations and propose to compensate them by taxing the volumes of sediment treated.

According to Benslafa et al. (2015), the sediments calcined around 700 °C can constitute a silico-aluminous pole, replacing on the one hand, cement up to 30% and on the other hand, replacing natural pozzolans in the concrete formulation. The rise in temperature transforms the stable clay structures into amorphous structures, ensuring the pozzolanic reactivity sought for the envisaged substitutions. Scordia et al. (2008) also show that sediments acquire pozzolanic qualities and, after mixing with hydraulic binders such as quicklime, can be used as a subgrade in road works.

Technically, using dredged sediments as clinkers for cement and similar applications is therefore possible. But the sediments can enter into competition with natural resources widely available to the cement manufacturer. Between the stages of dredging, dehydration, and transport of sediments to the cement factory, the economic viability of these sectors remains to be determined (Soleimani et al. 2023a).

### Dredged sediments for the manufacture of bricks and expanded aggregates

Along the same lines as in the manufacture of clinker, sediments can constitute substitutes for natural materials for the manufacture of bricks or tiles. Some have mineralogical compositions close to the materials generally used (clays, feldspar-type flux, and filler generally made of quartz). The heating temperature is around 1100 °C which corresponds to the sintering temperature. During the process, the periphery of certain minerals melts, which has the effect of welding the grains together via the vitreous phase produced. The use of sediments of various origins for the manufacture of bricks has been experimented by several authors (Chiang et al. 2008; Hamer and Karius 2002; Mezencevova et al. 2012). Most of the work concludes that the quality of the products produced is very satisfactory. Satisfactory results were obtained by Nguyen et al. (2023) at a laboratory scale on sediments from Texas (USA). These authors also highlight the savings in crushing, sieving, and washing provided by the use of sediments compared to conventional materials. Hamer and Karius (2002) encountered problems with efflorescence on bricks and the release of SO_2_ during their manufacture, but these disadvantages can be eliminated by gas treatment for the latter and the addition of certain additives for the former. The main obstacles to this sector would be, on the one hand, the manufacturing costs, a priori higher than “classic” bricks, and on the other hand, the image problem of the product made from dredged sediments. Cappuyns et al. (2015) show that the fears of potential buyers are linked to the reliability and durability over time of “sedibric” as well as the risk of transfer of the contaminants they may contain. Out of 434 people questioned, around 40% have a negative to very negative a priori. The authors conclude that there is a need for intense communication to make this sector viable.

Generally, products manufactured experimentally or on a pilot scale have the qualities required for the intended use, but the transition to an industrial scale very generally comes up against the financial cost and/or the difficulty of marketing on the market for products made from contaminated material waste. Under these conditions, most communities favor less technical solutions, requiring more limited sedimentary transformations such as the use of embankments.

### Dredged sediments as plant growth medium

When sediments are highly organic, their use in construction is limited since the organic material could negatively impact the quality of final products (Siham et al. 2008). Alternatively, these types of sediments when having low concentrations of pollutants could be employed as solid conditioners or plant-growing substrates, taking advantage of the properties of the material like enrichment of organic matter, micro and macronutrients and improvement of the water holding capacity (Kiani et al. 2021). The use of sediments in agriculture could reduce the dependence on fertilizers and contribute to finding new sources of phosphorous (Da et al. 2021). The phosphorus is limited on Earth, and more sustainable management promoting new recycling paths is required (Kiani et al. 2021). Several opportunities are offered to use dredged material in the plant industry, including the adoption in nurseries and soil conditioning of forest and agricultural lands or eroded fields (Renella et al. 2021). Several studies have been carried out on the possibility of using dredged sediments as a plant-growing substrate by co-composting with other wastes (Ferrans et al. 2021; Mattei et al. 2017; Todaro et al. 2018; Yao et al. 2009). The treated sediments showed no significant ecotoxicity or increased microbial diversity and allowed excellent plant growth. It can therefore be said that co-composting is an excellent sustainable option for reusing dredged sediment as a plant growth substrate. Ferrans et al. (2021) show that a main limitation of the growth was probably a lack of aeration of the sediments during the sampling and development of the experiment. The low aeration possibly caused a lack of available forms of N in the substrates, hindering the growth. Hence, sediments need to be pre-treated before using them to cultivate edible crops, or they could be employed to cultivate ornamental or bioenergy plants (Ferrans et al. 2021). Another application of dredged sediments in agriculture concerns the soil reconstruction (Technosol) and/or remediation. For instance, soil made using dredged material, manure, backwater sediment, and agricultural by-products was proposed to reduce the operational costs for the disposal of dredged sediment and enlarge the economic benefits of dredged sediment simultaneously (USACE 2015). Lee et al. (2007) stated that dredged sediment obtained from the mid-Atlantic coast can be used to create soils for a wide range of applications, such as brownfield redevelopment, gardening, and landscaping. However, the presence of heavy metals and phosphorus in the dredged material may constitute a real obstacle which may be the cause of a contamination of groundwater. Thus, there is a need to address these concerns prior to the beneficial use of dredged sediment for soil reconstruction/remediation. Fabbri et al. (2021) have shown that dredged sediments can be beneficial for the construction of technosols. However, the valorization of sediments as co-products of technosol construction requires analysis to ensure the safety of the raw materials. In addition, materials produced on-site or nearby minimize the cost and environmental impact of transport, so the involvement of local stakeholders in the management of the urban territory must be encouraged.

## Circular economic approach toward dredged sediments management

The concept of circular economy has further fueled this interest, as it promotes moving waste materials into useful resources through a sustainable approach (Nodehi and Taghvaee 2022). The traditional linear economy was first updated with the circular feedback loops which were originally initiated by Stahel in 1976 (Geissdoerfer et al. 2017). This began with the conventional 3R concept (Reduce, Reuse, and Recycle) (Almokdad and Zentar 2023). It was then followed by the real circular economy approach based on the 6R concept, which combines the three aspects of sustainability: economic, environmental, and societal through the principles of Reduce, Reuse, Recycle, Recovery, Remanufacturing, and Redesign (Almokdad and Zentar 2023). To implement the circular economy concept, these principles can be categorized into three main strategies: closing loops, slowing loops, and narrowing loops (Baldassarre et al. 2019). By example, in the construction sector, this involves creating circular material flow, increasing the lifetime of structures, and maximizing waste incorporation in construction.

**Fig. 4:**
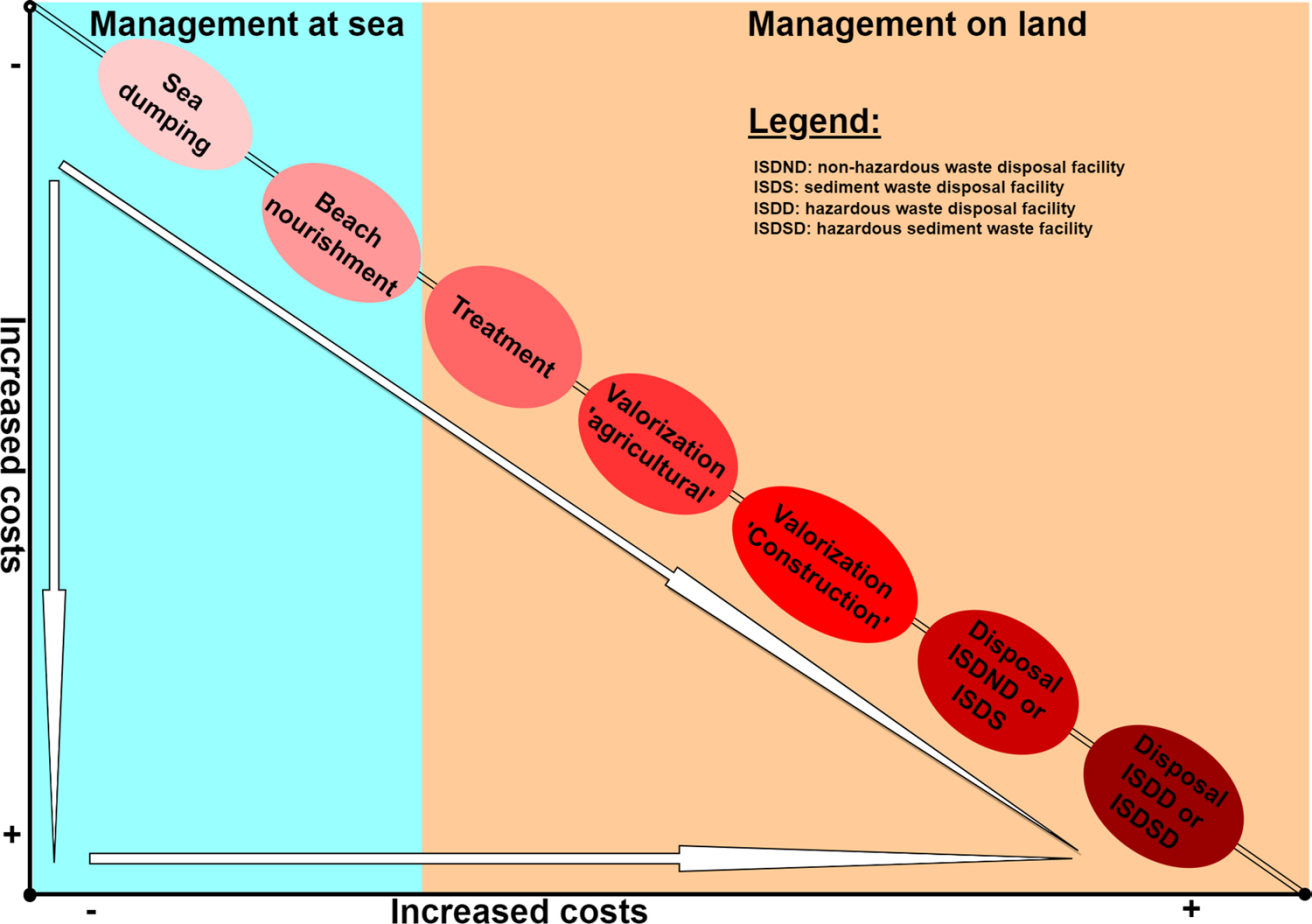
Variation in sediment management costs as a function of the pathway considered

The challenge of sediment management and reclamation involves controlling the economic and environmental costs of this management. The financial question to which the authorities in charge of managing port infrastructures and waterways must provide answers is very complex. It is difficult to establish a fixed costing for dredging operations, for the management processes of dredged sediments, for their treatment, and their recovery (Zeraoui et al. 2020). This difficulty is linked, on the one hand, to the fact that there is very little data on the economic aspect of dredging sediments, and on the other hand, to the significant disparities noted in the few data available in the literature. In general, the overall cost of dredging operations and sediment management depends mainly on the volumes dredged, the dredging techniques used, and the management methods (dumping, onshore management, disposal) (Fig. 4). The costs related to the management of dredged sediments depend on their management channels. Indeed, it appears that dumping at sea (when this solution is possible given the environmental constraints) remains the least expensive solution with an average cost estimated between 2 and 25 €/m^3^ (Svensson et al. 2022). Moreover, this solution represents more than 90% of the sediment management method (Harrington et al. 2022). The cost of onshore management is estimated at between 30 and 120 €/m^3^ (Harrington et al. 2022; Harrington et al. 2016) depending on the solution adopted. In addition to the impact of the management method on the cost disparity, the distance between the sediment collection point and the location where it will be reclaimed/disposed of has a large influence as well.

Various research studies (Achour et al. 2014; Beddaa et al. 2020; Benzerzour et al. 2017; Bianchini et al. 2019; Loudini et al. 2020a, b; Tessier et al. 2011; Wang et al. 2018a, b; Xu and Wu 2023; Yoobanpot et al. 2020; Zhao et al. 2022) developed process technology to recycled contaminated dredged sediments, and it seems that there is the potential of value proposition and economic viability of the products. The integrated life cycle assessment (LCA) (Soleimani et al. 2023a) and life cycle cost assessment (LCC) are also involved in economic analysis to provide insight for early-stage decision-making on the valorization of the dredged sediments (Soleimani et al. 2023b).

Furthermore, large-scale development of sediment-based products that are technically, economically, and ecologically feasible or able to compete in the current market of conventional products is still lacking (Norén et al. 2020) due to several obstacles that we must overcome.

## Bottleneck preventing bulk reuse of dredged sediments and recommendations for their management

### Technical and environmental barriers

Among the barriers cited in the literature, the fate of salts, heavy metals, and organic matter in contaminated dredged sediment is a central question. Due to their presence in contaminated dredged sediment, direct reuse in construction may lead to corrosion of reinforcement and chloride attack (Solanki et al. 2023). Therefore, relevant treatment techniques such as the desalinization, the stabilization of heavy metals, and organic thermal elimination should be applied before their reuse in construction in similar applications or other beneficial uses (Elghali et al. 2022). Thermal processes offer the advantage of mineralizing most organic compounds if they are brought to a sufficient temperature (otherwise fume treatment is necessary) (Wijesekara et al. 2007). They are also considered effective in trapping most trace elements (Corrochano et al. 2011; Wijesekara et al. 2007; Zhang et al. 2021). Ndiba and Axe (2010) show for example that a heat treatment at 700 °C, coupled with the addition of 2.5% phosphoric acid, transforms the speciation of Zn from a carbonate pole + association with iron hydroxides, into a pole made up of gahnite (ZnAl_2_O_4_), franklinite (ZnFe_2_O_4_), and hopeite (Zn_3_(PO_4_)_2_), very poorly soluble minerals. Hamer and Karius (2002) but also Cappuyns and Swennen (2009), however, highlight a significant leachable portion of certain elements such as arsenic (As), vanadium (V), and chrome (Cr) from bricks made from contaminated sediments. In laboratory tests, Zhang et al. (2009) highlight the loss by volatilization of all of the mercury contained in the initial sediment from 500 °C and note the loss of more than 10% of Cd at 800 °C. Smoke treatment may therefore be necessary depending on the materials treated. Leaching tests on sediments stabilized with hydraulic binders in the case of concrete manufacturing generally show low levels, consistent with the thresholds set by the various authorities (Said et al. 2015; Silitonga 2018). This trapping may be due to the formation of minerals which incorporate the contaminants into their network or to “physical” trapping due to the precipitation of these minerals around the contaminants (notably organic) (Hale et al. 2017). In the case of landscape mounds and the use of unmodified dredged sediments in soil construction, progressive oxidation can be the cause of greater mobility of contaminants. Today, the industrial sector is demanding less invasive, cheaper and cleaner means of treating salty waste such as dredged sediments. The objective is to reach the thresholds for discharge into the natural environment or to declassify sediments in storage facilities and thus reduce management costs. In the best case, sediments can be revalorized, for example, once treated, dredged sediments can be used as spreading sludge or technosol (Macia et al. 2014) or in road subgrade construction (Abriak et al. 2023).

### Socio-economic barriers

It is often a problem of the degraded image conveyed by waste. Even if waste can be recovered, the status of waste remains negatively connoted, particularly among populations and local residents’ associations. When a project owner carries out a dredging project, in the case where the quality of the sediments allows him to consider recovery, he must carry out close consultation with potential buyers of the materials; during this consultation, the “reputation” of the materials in question is essential to set up sectors, particularly in the case of market competition (cases where spreading is already practiced with sewage sludge, cases where quarries need backfill materials, etc.). This reputation can be degraded by the status of waste. This can harm the possibility of valorizing sediments with local stakeholders and associations, particularly during preliminary negotiations (spreading plan) or public inquiries linked to the water law file, for example. Thus, the waste status of sediments does not improve their social acceptability among stakeholders likely to reuse them (farmers, quarrymen, etc.). The possible commercial value of the material does not benefit from a positive impact linked to this status either, which can slow down private investors.

### Regulatory barriers

Regulations procedures are too cumbersome and a lack of security is observed in the sectors. The idea frequently expressed is that the waste status of sediments managed on land induces numerous regulatory procedures (AIDA 2014, 2020), often long and uncertain in terms of obtaining administrative authorizations, and always synonymous to additional costs for the management project. In other words, it is suggested that everything would be simpler from a regulatory point of view if the dredged sediments managed on land were not classified as wastes (for example, no dangerous properties to characterize, no study of impact to be achieved, etc.).

Once these technical constraints have been mastered, it appears essential to master other criteria as recommendations in order to guarantee the success of the dredged sediment reuse sectors:

- propose production costs proportionate to the challenges and considering competition with often cheap raw materials;
- ensure the existence of a market capable of absorbing production, considering possible lobbying if the recycled product competes with raw materials;
- ensure the sustainability of the sector through good knowledge of the “deposition” of dredged sediments (quantity and quality);
- master the legal aspects: responsibility of the waste producer, legal operating authorization, and method of reusing materials;
- ensure the social acceptability of the material produced which may suffer from its image as potentially contaminated waste.

## Conclusion

The dredging of sediments in marine and estuarine areas constitutes an essential activity for the proper functioning of these structures. In Europe and in France, the improvement of diagnostics and knowledge, the development of greater sensitivity of populations, port managers, and stakeholders to the environmental impacts of dredging activities, as well as the progressive lack of land for the final storage on land of dredged sediments have called into question management practices. Therefore, new onshore management strategies are being sought for dredged sediments. The problems posed are more or less glaring depending on local particularities but overall, all encourage the reuse of dredged sediments in compliance with European directives and French laws in this area. Concerning this potential reuse, dredged sediments have to be brought back to the land where they are considered waste. French waste regulations aim to protect people and the environment. To do this, it uses tools and methods to assess increased risks. It requires the establishment of traceability of recovered materials and defines the responsibilities of the actors. In this framework, it opens the door to many ways of using sediments, but with the necessity to the project owner to prove the validity and safety of the material. In practice, the organization of the sector is considered cumbersome by the various stakeholders. Above all, project owners and design offices do not always know where to set the limits of the exercise aimed at defining the safety of materials: the studies requested can prove costly, with no guarantee of validation of the results by the state services, which can discourage initiatives. The valorization of dredged sediments therefore encounters problems of uncertainty at several levels (but ultimately all linked to the heterogeneity of the sediment): quality of the sediment, validation by State services, and sustainability of the work.

The analysis of potential sectors of beneficial reuse of dredged sediments in the South of France (clinkers for cement and similar applications, manufacturing of bricks and expanded aggregates, medium of plant growth) was also discussed. It has highlighted one strict impossibility from a normative point of view; for example, the use of sediments for construction, due to their often too high content in organic matter, salts, and heavy metals can be problematic without any pretreatment. For the other recovery sectors envisaged, the analysis does not reveal a ban on the use of dredged sediments, but attention must be paid to the chloride, sulfate, and sulfide contents in the leachates and/or solids, for applications as aggregates for concrete with metal reinforcements. In addition, the materials used must meet physical, chemical, or mechanical criteria that raw sediments do not present, but which can be obtained by treatment or mixing with other materials. For this, technologies have emerged, aimed at prior decontamination of dredged sediments with a view to their beneficial reuse.

However, if the products manufactured experimentally or on a pilot scale have the required qualities, the transition to an industrial scale very generally comes up against the financial cost and the constraints induced by the manufacture of products from a deposit of heterogeneous sediments, sometimes contaminated. Once the technical constraints have been mastered, it appears essential to satisfy other criteria such as the existence of a market capable of absorbing the production and the social acceptability of the material produced in order to guarantee the success of the recovery sector. Furthermore, there are numerous coordinated interdisciplinary articles on research in the areas of innovative technologies related to the treatment of sediments for reuse but beyond the scope of our study. The bibliography cited here is limited to filters applied in the Web of Science database and may have missed some good journal references. On this basis, environmental studies concerning the treatment of sediments in geotubes and the manufacture of technosols based on dredged sediments are emerging themes in this field of research. We can expect more research and publications in the coming days on the possibilities of incorporating treated sediment as co-products of technosol manufacturing.

## Data Availability

Not applicable.
